# Bisphenol-A and Female Fertility: An Update of Existing Epidemiological Studies

**DOI:** 10.3390/jcm11237227

**Published:** 2022-12-05

**Authors:** Konstantinos Stavridis, Olga Triantafyllidou, Maria Pisimisi, Nikolaos Vlahos

**Affiliations:** 1 2nd Department of Obstetrics and Gynaecology, Aretaieion Hospital, University of Athens, 11527 Attica, Greece; 2 School of Medicine, National and Kapodistrian University of Athens, 11527 Athens, Greece

**Keywords:** bisphenol-A, chemical disruptor, fertility endometriosis, PCOS, in vitro fertilization

## Abstract

Humans interfere with a variety of endocrine disruptors on a daily basis, which may result in adverse health effects. Among them, Bisphenol-A (BPA) is the most debated endocrine disruptor, despite being widely studied, regarding its effects on fertility. The aim of this review was to investigate the interrelation of BPA and female fertility. PubMed (Medline) was searched from 2013 until 2022 to identify epidemiological studies that report the association of BPA with fertility parameters, in vitro fertilization (IVF) outcomes, polycystic ovarian syndrome (PCOS) and endometriosis. Regarding general fertility, most studies report an inverse association between BPA and ovarian reserve markers, namely antral follicle count (AFC) and anti-Müllerian hormone (AMH). The BPA and estradiol (E2) levels did not correlate significantly in the majority of studies. No definite conclusions can be reached regarding BPA and IVF endpoints or endometriosis. Lastly, most studies report higher prevalence of PCOS in women with higher BPA concentrations, although no casualty has been proven. Although most studies fail to reach definite conclusion regarding the impact of BPA on fertility, there is accumulating evidence suggesting a negative role of BPA in female reproductive health.

## 1. Introduction

The urbanization and industrialization of modern societies have undoubtedly led to the degradation and pollution of the environment, posing further threats to human health [1]. Individuals are exposed to an extensive variety of modern chemical substances daily, such as phthalates, bisphenol-A (BPA), triclosan (TCS), parabens and many more, all identified as endocrine-disrupting chemicals (EDCs). EDCs are substances found in a variety of products used daily that interfere with endogenous hormonal systems at various levels, resulting in adverse health effects [2]. During the past decade, global health communities have succeeded in improving and protecting maternal and child health to a great extent, focusing primarily on reproductive health [3,4]. Nevertheless, infertility remains a major medical, psychosocial, and economic challenge of the present era. In a National Survey of Family Growth (NSFG) conducted between 2015 and 2019, the percentage of married women aged 15–49 who were infertile ranged between 12.6% and 26.8% [5]. The leading causes of female infertility seem to involve certain tubal and uterine disorders, as well as ovarian and endocrine diseases, such as polycystic ovarian syndrome (PCOS) and endometriosis [6].

Bisphenol-A (2,2-bis(4-hydroxyphenyl) propane, CAS No. 80-05-7) is one of the most extensively studied and debated EDCs. To date, several studies have been carried out, highlighting the role BPA plays in the reproductive capacity of women. This substance is mainly used in the plastic industry to manufacture numerous consumer products, including epoxy resins, food and drink containers, baby bottles, polycarbonate plastics, thermal receipt paper, personal care products, dental sealants, etc. Although the main source of exposure to BPA is assumed to be oral ingestion [7], lately, scientific interest has turned towards the transdermal route of exposure [8]. 

BPA seems to have a particularly high affinity for estrogen receptors (ERα and β), as it disposes of phenol group, such as estradiol (E2), and therefore exhibits estrogen-mimicking behavior [9,10]. This suggests that such a chemical compound could potentially arouse estrogen function and contribute to the pathogenesis of various diseases of the female reproductive system by disrupting the hypothalamic pituitary–gonadal axis. Moreover, BPA has the capacity to inhibit androgen function by binding androgen receptors (AR). Additionally, several studies have illustrated the ability of BPA to bind G-protein-coupled estrogen receptors (GPER30) and, therefore, interfere with estrogen-activated signaling pathways [11,12,13].

Due to its reproductive toxicity, BPA usage has been restricted globally over the past few years. Instead, certain BPA analogues were introduced in baby bottles, infant formula packaging and other plastic materials; bisphenol S (BPS; bis(4- hydroxyphenyl sulfone) and Bisphenol F (BPF; 4,4′-dihydroxydiphenyl methane) are two of the most commonly used analogues of BPA.

Our study aims to critically review the current epidemiological studies evaluating the association of serum and/or urinary BPA levels and female fertility, mostly focusing on in vitro fertilization (IVF) outcomes and on infertility-related reproductive disorders, namely endometriosis and PCOS.

## 2. Materials and Methods

PubMed (Medline) was searched to identify observational studies published between May 2013 and May 2022 that were related to BPA and fertility. We focused on articles published between 2013 and 2022 to expand on previous reviews on the same topic [14,15]. The search was limited to English papers and human studies. The following search terms were combined: ‘Bisphenol-A’, ‘BPA’, ‘female fertility’, ‘female fecundity’, female reproduct*’, ‘ovary’, ‘oviduct’, ‘uterus’, ‘HPO’, ‘anti-Mullerian hormone (AMH), ‘FSH’, ‘estradiol’, ‘ovarian reserve’, ‘AFC’, ‘oocytes retrieved’, ‘fertilization rate’, ‘implantation rate’, ‘pregnancy rate’, ‘live birth’, ‘PCOS’, ‘Endometriosis’. We further hand-searched the citations of the retrieved eligible papers to identify additional publications that might have been missed during the initial search. Cohort, case-control and cross-sectional studies reporting the association of BPA with female fertility, PCOS and endometriosis were included. Studies reporting in vitro experiments, which may fail to replicate the conditions of cells in an organism or predict in vivo behavior, were excluded.

From each study, the following information was abstracted: first author, publication year, study location, study period, sample size, female age, and type of study (Table 1).

## 3. Results

### 3.1. Bisphenol-A and Fertility

The association between BPA and fertility impairment constituted a matter of discussion in several studies (Table 2). The researchers examined the effect of BPA either on ovarian reserve parameters or on IVF outcomes, both of which defined the success of an IVF cycle. Furthermore, some studies compared the levels of BPA between fertile and infertile women with the aim of finding a link between BPA levels and fertility, although in this category no causality could be established. 

In 2013, Souter et al. investigated the relationship between urinary BPA levels and ovarian response during IVF among women undergoing infertility treatments from the EARTH cohort. The researchers found a decrease in antral follicle count (AFC) with higher BPA levels (*p* < 0.001). However, no association between BPA and FSH or ovarian volume (OV) was reported [19]. Similarly, Zhou et al. reported that a unit increase in BPA is associated with a statistically significant decrease in AFC. The researchers also noted that BPA is negatively associated with anti-Müllerian hormone (AMH) and day-3 FSH, but no statistical significance was found [27]. Two recent studies confirmed the aforementioned data [47,50]. Czubacka et al., in particular, found a negative association of high BPA levels with AFC and AMH [47]. Similarly, Lin et al. reported a lower AFC count in the group with high BPA levels, although no statistical significance was reached [49]. However, with regards to these associations, two studies reported different conclusions. A Korean cross-sectional study reported higher BPA levels in women with diminished ovarian reserve (DOR), although no significant correlation of EDCs with AFC or OV was shown [39]. Furthermore, Li et al. reported a positive association of BPA with LH and no association with AFC or FSH [44]. All of the above studies indicate that BPA could adversely affect oocyte viability. However, they are not designed to identify the exact mechanisms by which these alterations could be established.

Regarding the association between BPA and E2 levels during IVF, most studies report no association [23,35,41,47]. However, some studies reported a possible negative correlation between BPA levels and peak E2 [49,51], whereas one study, in 2015, reported a positive association between BPA and E2 levels [24]. More specifically, Lin et al. analyzed BPA concentrations in normo-gonadotropic infertile patients who underwent their first IVF-ET cycle, reporting lower peak E2 levels in the high-BPA-exposure group, although this result was not statistically significant, possibly due to the small sample size of the study [49]. Yenigül et al. also observed lower E2 levels in women with higher BPA exposure; again, this association was not statistically significant [51]. On the other hand, in the study by Miao et al. a positive association between urine BPA and E2 was observed with borderline significance among women in the BPA-exposed group (*p* = 0.05) [24]. These findings suggest that BPA exposure may interfere with E2 production during gonadotropic stimulation. Lastly, two research groups that examined the BPA levels in adolescents indicated an inverse association between BPA levels and E2 in young females [38,46], which clearly confirmed the aforementioned data regarding the interference of BPA with E2 production.

Overall, seven studies have investigated the association between serum or urine BPA concentrations and various IVF outcomes [23,35,41,45,49,50,51]. Most of them reported the absence of a statistically significant association between high BPA concentrations in various human fluids and IVF outcome measures, namely fertilization, clinical pregnancy, and live birth rates [23,35,41,51]. However, Nazli Yenigul et al. found a lower clinical-pregnancy and live-birth probability, as well as decreased embryo quality, in women undergoing intra-cytoplasmic sperm injection (ICSI) with higher serum and follicular-fluid BPA levels [51]. Additionally, Aftabsavad et al. observed lower clinical pregnancy rates and total embryo and oocyte quality in the group of women with unhealthy lifestyle habits (group 2), among whom the BPA levels also appeared to be significantly higher in comparison to the group with healthy lifestyle habits (group 1) [50]. Lin et al. note a statistically significant association between high urinary BPA levels and decreased embryo implantation, oocyte retrieval and maturation rates [49]. Moreover, Radwan et al. report a statistically significant decrease in implantation rates and MII oocyte counts in women who present higher urine BPA concentrations [45]. 

In addition, several studies assessed the levels of BPA in populations with different infertility impairments. Pednekar et al. reported higher BPA levels in an infertile population compared to healthy individuals [32], whilst Özel et al. found that women with premature ovarian insufficiency had increased serum BPA concentration [33]. Arya et al. found that individual BPA levels are not associated with infertility, but a combination of BPA, benzophenone-3 (BP-3) and TCS exposure may be positively correlated with infertility [37]. Nevertheless, it should be highlighted that a major limitation of this study is the lack of a medical confirmation of impaired fecundity, as the study relied on self-reported infertility. Caserta et al. also found higher BPA levels in infertile women, as well as a positive association between BPA and Era-, ERb-, AR-, AhR- and PXR-gene expression [18]. 

Finally, in 2020, a research group evaluated the relationship between bisphenols and thyroid function, since hyperthyroidism is known to affect fertility. The researchers found that only urine BPC is associated with high TSH levels and may therefore interfere with normal fertility [40].

### 3.2. Bisphenol-A and PCOS

PCOS constitutes one of the most common endocrine diseases among women of reproductive age [53]. PCOS is associated with endocrine, metabolic and reproductive health implications, including anovulation, infertility, hyperandrogenism, obesity, hyperinsulinism, increased risk of type-2 diabetes and cardiovascular disease [54,55,56]. Although the exact etiology and pathophysiological mechanisms of this syndrome remain enigmatic, many studies have highlighted the potential role of ethnic origin, geographic location, lifestyle and environmental factors in the pathogenesis and/or clinical manifestation of PCOS [57,58]. Among environmental factors, BPA has raised great concern regarding its association with PCOS, mainly due to its estrogen-like actions. 

Between 2013 and 2022, ten human studies were identified which examined the association of bisphenols, particularly BPA, with PCOS (Table 3). In 2013, Tarantino et al. concluded that high serum BPA levels in PCOS women are associated with higher grades of insulin resistance, hepatic steatosis, free-androgen index (FAI), inflammation and spleen size [16]. Two later studies, which measured serum BPA levels in healthy and PCOS women, found that the BPA levels were higher in women with PCOS compared to the control group [22,25]. In particular, Akin et al.’s study, which evaluated 173 female adolescents, reported statistically significantly higher mean BPA levels in PCOS women compared to healthy women (1.1 ng/mL vs. 0.8 ng/mL), as well as a statistically significant correlation between BPA and androgen levels [22]. More recent publications confirm the aforementioned association of high BPA levels and PCOS [29,31,36,48,52]. In particular, a research group from Poland reported significantly higher BPA levels in women with PCOS compared to the control group (*p* = 0.035) and a positive correlation of BPA with FAI and serum total testosterone (TT) [31]. The correlation reported by previous studies between high BPA and androgen levels may suggest a potential contribution of BPA to the ovarian hyperandrogenism usually seen in women with PCOS. Lazúrová et al. found analogous results regarding high BPA levels and PCOS associations; however, the research group reported a negative association between U-BPA levels and TT, free testosterone (FT) and FAI, suggesting controversial results concerning BPA and androgen levels [52]. Akgul et al., in 2019, found that BPA is associated with polycystic morphology on ultrasound in adolescent females with PCOS, but no association with obesity, androgen levels or other metabolic parameters was reported [36]. Finally, a recent study by Jurewicz et al., who evaluated the association between PCOS and BPA and its analogues, namely BPS and BPF, found that, in women with PCOS, only BPS was significantly higher compared to the control group (*p* = 0.023) [48]. However, in disagreement with the aforementioned studies, two research groups found no association between BPA levels and PCOS [21,30]. Vagi et al. reported no association of creatine-adjusted urine BPA levels with PCOS. However, this study had several limitations, particularly the small sample size and the inadequate confounding adjustment for BMI and age [21]. Similarly, Gu et al., in 2018, did not find a significant relationship between PCOS and urinary BPA either in an unadjusted binary logistic regression model or in a model adjusted for potential confounders [30]. All of the above data, although controversial, highlight that PCOS development may be strengthened by high BPA levels in female populations, mainly due to the potential contribution of BPA to ovarian hyperandrogenism.

### 3.3. Bisphenol A and Endometriosis

Endometriosis is commonly defined as the presence of endometrial glands and stroma outside the endometrial cavity, primarily on the pelvic peritoneum, ovaries, rectovaginal septum and, in rare cases, on the diaphragm, pleura and pericardium [59]. This is a common disease entity, as, according to epidemiological studies, it affects roughly 6–10% of reproductive-age women and girls globally [60]. Despite its high prevalence, endometriosis remains an enigmatic disease, as its pathogenesis has not yet been established. Many factors have been implicated at different times, including retrograde menstruation, genetics and immunity; however, in the past few decades, questions have arisen concerning the possible role of EDCs in the pathogenesis of endometriosis. It is generally considered a benign condition and can be asymptomatic; however, this perplexing disease is frequently responsible for symptoms such as chronic pelvic pain, dyspareunia, dysmenorrhea and, most significantly, infertility, which constitutes a serious consequence. To establish a definite diagnosis, surgical visualization is required [61].

According to our research, only a handful of population-based studies have been carried out to investigate the possible association between BPA exposure and the occurrence of endometriosis in humans (Table 4). All seven studies assessed urine specimens and some considered creatinine adjustment [17,20,34,42,43]. Interestingly, the results of these studies were not in agreement with each other.

In 2013, the ENDO study [17] aimed to examine the association of urine BPA with endometriosis by establishing two cohort groups, an operative and a population cohort. The latter group was screened through standardized pelvic magnetic-resonance imaging (MRI) for the assessment of the disease and no evident association with BPA exposure was found. As far as the operative group is concerned, again, no statistically significant correlation was found between elevated BPA levels and the diagnosis of endometriosis. Similarly, in 2019, Moreira Fernandez et al. found no association between endometriosis and urine BPA concentrations. In this case-control study, the participants were Brazilian women aged 18–45 and the diagnosis of the disease was confirmed or excluded by laparoscopy with a visual inspection of the pelvis and biopsy of suspected lesions [34].

In 2014, Upson et al. [20] collected data from the Women’s Risk of Endometriosis (WREN) study and did not observe a statistically significant correlation between total urinary BPA concentrations and endometriosis overall, after adjusting for possible confounders. Hence, the report that higher BPA levels may increase the risk of non-ovarian pelvic endometriosis is intriguing (*p* > 0.005).

By contrast, both Simonelli et al., in 2016 [26], and Rashidi et al., in 2017 [28], reported a significant correlation between endometriosis and BPA exposure. Simonelli et al. conducted a prospective case-control study that included 144 women from Southern Italy and, meanwhile, assessed the possible occupational and environmental exposure, concluding with the finding of statistically significantly higher urinary BPA levels in the patient group than in the control group and highlighting the need for further investigation regarding occupational exposure and certain lifestyle factors [26]. Rashidi et al. studied the cases of 100 women from Iran, taking into consideration possible confounding factors and indicated a positive association between urinary BPA concentrations and the presence of ovarian endometriomas [28].

The main objective of Peinado et al. (2020) [43] was to evaluate the concentrations of BPA and its analogues (BPS and BPF) regarding the levels of thiobarbituric-acid-reactive substances (TBARS) and the risk of endometriosis in women of reproductive age. This case-control study reports a possible relationship between inadvertent exposure to bisphenols and the risk of endometriosis and suggests a potential role of oxidative stress in the endocrine-disruptive effects of BPA in these women. Wen et al. (2020) [42] studied two hundred twenty (220) women investigating a possible association between urinary BPA concentrations and matrix metalloproteinase (MMP2, MMP9) expressions and the risk of different endometrioma (EM) subtypes. It is noteworthy that matrix-metalloproteinase activity is thought to be essential in the early phases of endometriosis development. Although this study reported no association between creatinine-adjusted urinary BPA concentrations and EMs overall, a statistically significantly positive correlation emerged when evaluating urinary BPA concentrations apropos of peritoneal EMs. This finding is partly consistent with the results of the study by Upson et al. [20]. In addition, a positive association was found between urinary BPA levels and serum MMP2 and MMP9 levels.

## 4. Discussion

Recent studies have shown that EDCs, and particularly BPA, interfere with the female genital tract and may be associated with infertility, as well as infertility-related diseases, namely PCOS and endometriosis. Numerous studies suggest that, as an endocrine disruptor, BPA binds with Era, ERβ and transmembrane ERs, resulting in the induction of alternative estrogen signaling and the distortion of hormonal balance. Through binding, BPA either mimics (agonistic effect) or blocks (by inhibiting the aromatase activity) natural human estrogens, consequently disturbing the development, regulation and endocrine control of the female genital tract. The two-way estrogenic action of BPA could explain the diversity of our results, according to which one study found a positive correlation of BPA with E2 [24], while others reported an inverse correlation of BPA with E2 levels [38,46,49,51]. Nonetheless, most of the studies revealed no statistically significant correlation [23,35,41,47].

In addition, BPA is known to have adverse effects on the process of oocyte maturation and meiotic-cell-division machinery. Thus, BPA can impair oocyte survival, inhibit follicular growth and diminish ovarian reserves [19,41]. To confirm these actions, the epidemiological studies reported in this review examined the association of BPA with well-known ovarian-reserve markers, namely AFC and AMH. Overall, the studies reported an inverse correlation between ovarian predictors and BPA levels. On the other hand, the data reporting an association of day-3 FSH and BPA levels remain controversial. Furthermore, the estrogenic action of BPA could affect implantation and fertilization rates, which are highly modulated by estradiol and progesterone. Diminished oocyte maturity, along with possible chromosomal abnormalities, could also explain the impairment with regards to pregnancy rates. Indeed, various studies presented in the current review correlated high BPA levels with different IVF outcome measures, such as oocyte quality, embryo quality, fertilization rates, implantation rates and clinical pregnancy rates. Different endpoints were examined by each study, which makes definite conclusions difficult. Overall, the studies indicate that high BPA levels could affect IVF success in various stages, in either oocyte maturation or fertilization and implantation rates. Clinical pregnancy rates are also reported to be highly affected.

It is worth mentioning that, recently, several studies have highlighted the protective role of soy-based foods against the negative effect of BPA on IVF outcomes. Similar to BPA, soy-food components are characterized by estrogenic activity and, therefore, have the ability to interfere with BPA-induced effects on DNA methylation [62,63].

Furthermore, several studies, mainly cross-sectional and case-cohorts, revealed higher BPA levels in women with fertility impairment compared to control groups, which suggests an association between high BPA levels and infertility. However, these studies, as well as epidemiological studies in general, inevitably hamper the interpretation of causality.

Interestingly, Milczarek-Banach et al. conducted the only study in which the effect of different bisphenol analogues on thyroid function was examined. The researchers reported that urine bisphenol-C (BPC) is negatively associated with thyroid volume [40]. Thyroid-hormone levels and TSH are known to affect IVF outcomes [64] and research concerning BPA levels and thyroid-function association is scarce. Hence, more studies relevant to this topic are required.

Growing evidence suggests that BPA exposure may be involved in the occurrence of PCOS. Indeed, several mechanisms explain how BPA can contribute to the pathogenesis of PCOS. Firstly, BPA can directly increase the synthesis of ovarian androgen levels in theca cells by increasing the levels of certain steroidogenic enzymes, such as P450c17, P450scc and StAR [65]. Secondly, BPA can also interact with granulosa cells by augmenting FSH-stimulated progesterone synthesis [66]. At the same time, BPA also has the capacity to displace sex hormones from sex-hormone-binding globulin (SHBG), which could also contribute to higher free-androgen levels [67]. Furthermore, BPA has an effect on obesity and metabolism by inhibiting the release of adiponectin, a protein that acts against the development of metabolic syndrome [68]. Furthermore, it has been suggested that PCOS is a pro-inflammatory state; inflammation is thought to play a vital role in the pathogenesis of PCOS [67]. Finally, BPA can directly interfere with ß-cells by increasing insulin production. The consequent hyperinsulinemia increases insulin resistance in peripheral tissues, which also contributes to the disarrayed metabolic profile of PCOS [69]. Most of the studies analyzed in our review reported a statistically significant association between PCOS and high concentrations of BPA in various human fluids (serum and urine specimens) due to increasing androgen levels and serum TT. Nonetheless, the retrospective case-control and cross-sectional designs of the existing human studies, which determine prevalence and do not unravel causal relationships, need to be addressed. It remains unclear whether the observed association indicates that BPA contributes to the development or presentation of the disease or whether the endocrine profile of PCOS alters the storage and clearance of this metabolite, leading to increased concentrations in different human fluids. Additionally, a strong correlation between elevated BPA exposure and hyperandrogenism in the setting of polycystic ovary syndrome was described by various authors [16,22,31], although it needs to be acknowledged that elevated androgen levels down-regulate the activity of UDP-glucuronosyltransferase, inducing a decrease in BPA clearance and an increase in its urine and blood concentration [70].

Certainly, accumulating evidence over the last decade strongly suggests a link between EDC exposure and the subsequent development of endometriosis. Notwithstanding the lack of knowledge of the mechanisms associated with the institution of the disease, it is well documented that ectopic-lesion growth is an estrogen-dependent process [71] commonly characterized by progesterone resistance and inflammatory disorder [72]. The epidemiological data on the effect of BPA exposure on the establishment of the disease are limited and controversial, with an increased endometriosis risk reported by some authors but no association by others. It is paramount that we develop a better understanding of the possible contribution of BPA exposure on the incidence and pathophysiology of endometriosis. Undoubtedly, it should be pointed out that there is no biomarker for endometriosis, which constitutes a barrier for research at the population level. Furthermore, the diagnostic method poses a certain challenge (surgical visualization with/without histologic confirmation vs. self-reported disease) that could possibly affect the validity of this disease in these studies. Altogether, our inconsistent findings suggest that further observational studies are required to elucidate the role of BPA in the pathogenesis and manifestation of endometriosis.

There are possibly numerous factors contributing to the divergent results of our study. Firstly, previous researchers have used a variety of specimens (urine, blood, follicular fluid) for exposure assessment. Most studies indicate that urine-BPA measurement may be more efficient in determining exposure to this compound, because it probably reflects more long-term exposure in comparison to blood or serum measurements [35,52]. It is well known that in humans, after ingestion, BPA is rapidly metabolized and almost completely excreted in urine as the glucuronide conjugate because the free form of BPA is insoluble in water [73]. Moreover, blood measurement may pose a risk of contamination during sample collection [29]. Secondly, most of the studies were designed with a single-spot urine measurement of BPA. However, BPA is a non-persistent EDC with a urinary elimination half-time of less than 6 h [21]. Therefore, to avoid the misclassification of BPA exposure due to its quick metabolism, future studies are encouraged to measure 24-hour urine BPA. Furthermore, although some studies measured urine creatinine to evaluate the daily exposure for BPA, most studies did not. As highlighted by Wen et al.’s study in 2020, before creatinine adjustment, the absolute values of BPA at different time points varied significantly. However, after creatinine adjustment, urinary BPA levels at different time points showed high consistency, suggesting that the creatine-adjusted urine measurement of BPA may be more reliable [42]. Another issue that raises concerns is the fact that in all the studies, BPA was measured after the onset of the disease and not before. Thus, exposure to this metabolite was not disclosed at the time of the genesis of the disease. Lastly, although most of the studies adjusted in their statistical analysis for known confounders, such as BMI and age, most of them did not take into consideration simultaneous co-exposition to many other EDCs, which may also contribute to infertility impairment. The findings by Arya et al. are crucial and indicative; BPA was found to not be correlated to fecundity when studied individually, although the simultaneous study of three EDC (e.g., BPA, BP-3 and TCS) confusing factors found a statistically significant relationship [37].

## 5. Conclusions

In this appraisal of the recent literature, evidence indicates that BPA might play a vital role in the pathogenesis of female infertility. Our review indicated that BPA is negatively correlated with certain IVF parameters and endpoints, as well as PCOS. Regarding the association of BPA with endometriosis, recent data failed to reach definite conclusions. Although there is a variety of experimental studies in the literature concerning BPA and infertility, human studies are still limited. More insight would be gained by a consistent human-fluid analysis of BPA, such as urine vs. serum and, possibly, the use of biobanks to assess long-term consequences. Despite the lack of sufficient data to reach a consensus with regards to the possible role of BPA in the onset of certain gynecological manifestations, precautionary actions against excess exposure to this metabolite are strongly encouraged.

## Figures and Tables

**Table 1:** Characteristics of studies included.

Author, Year	Location	Study Period	Sample Size	Female Age (Mean)	Type of Study
Tarantino et al., 2013 [16]	Naples, Italy	November 2009–October 2011	60	27.7 ± 6.8	Cross-sectional
Buck Louis et al., 2013 [17]	Salt Lake City, Utah and San Francisco, California, USA (ENDO study)	2007–2009	600	32.0± 6.8 (Operative cases) 33.6 ± 7.1 (Operative control) 33.1 ± 8.3 (Population cases) 32.1 ± 7.8 (Population control)	Matched cohort
Caserta et al., 2013 [18]	Rome, Italy	January 2009–April 2010	155	35.3 ± 0.4 (cases) 34.8 ± 4.6 (control)	Cross-sectional
Souter et al., 2013 [19]	Massachusetts, Boston, USA (EARTH study)	November 2004–October 2010	209	36.1 ± 4.2	Prospective cohort
Upson et al., 2014 [20]	Seattle, Washington, USA	April 1996–March 2001	430	N/A	Cross-sectional
Vagi et al., 2014 [21]	Los Angeles, California, USA	March 2007–May 2008	102	28.12 (cases) 31.84 (control)	Case-control
Akin et al., 2015 [22]	Kayseri, Turkey	January 2011–August 2012	173	15.4 ± 1.2	Cross-sectional
Mínguez-Alarcón et al., 2015 [23]	Massachusetts, Boston, USA (EARTH study)	November 2004–April 2012	256	36.0 ± 4.3	Prospective cohort
Miao et al., 2015 [24]	Shanghai, China	N/A	356	N/A	Retrospective cohort
Vahedi et al., 2016 [25]	Tehran, Iran	N/A	124	N/A	Case-control
Simonelli et al., 2016 [26]	Naples, Italy	N/A	128	N/A	Case-control
Zhou et al., 2016 [27]	Shandong, China	June 2014–October 2014	268	27	Cross-sectional
Rashidi et al. 2017 [28]	Tehran, Iran	N/A	100	32.22 ± 5.34 (cases) 33.20 ± 5.46 (control)	Case-control
Rashidi et al. 2017 [29]	Tehran, Iran	September 2013–September 2014	102	29.80 ± 7.02 (cases) 32.96 ± 5.58 (control)	Case-control
Gu et al., 2018 [30]	Shandong, Zhejiang province and Shanghai, China	N/A	123	30.5 (cases) 29.8 (control)	Case-control
Konieczna et al., 2018 [31]	Gdańsk, Poland	January 2016–December 2017	186	26.9 ± 5.2 (cases) 28.2 ± 5.7 (control)	Cross-sectional
Pednekar et al., 2019 [32]	Mumbai, India	May 2015–August 2015	79	28.4 ± 4.4 (cases) 26.8 ± 3.8 (control)	Pilot case-control
Özel et al., 2019 [33]	Ankara, Turkey	June 2017–October 2017	60	29.6 ± 6.6 (cases) 29.5 ± 5.2 (control group)	Cross-sectional and case-control
Avelino Moreira Fernandez et al., 2019 [34]	Minas Gerais, Brazil	N/A	52	36 (cases) 34 (control)	Case-control
Kim et al., 2019 [35]	Seoul, Korea	August 2013–July 2014	146	N/A	Prospective cohort
Akgül et al., 2019 [36]	Ankara, Turkey	March 2016–March 2018	95	15.62 ± 1.29 (cases) 16.04 ± 1.59 (control)	Case-control
Arya et al., 2020 [37]	USA (NHANES study)	2013–2014 and 2015–2016.	895	31.8± 8.1	Cross-sectional
Pollock et al., 2020 [38]	USA (NHANES), Canada (CHMS)	2013–2016 for U.S. population (NHANES) 2012–2015 for Canadian population (CHMS)	5100	N/A	Cross-sectional
Park et al., 2020 [39]	Seoul, Korea	September 2014–November 2014	307	36.8 ± 4.4	Cross-sectional
Milczarek-Banach et al., 2020 [40]	Warsaw, Poland	October 2017–May 2019	180	24 ± 3	Cross-sectional
Shen et al., 2020 [41]	Zhejiang Province, China	September 2013–October 2016	351	31 ± 3	Prospective cohort
Wen et al. 2020 [42]	Wuhan, China	October 2017–December 2018	220	N/A	Case-control
Peinado et al. 2020 [43]	Granada, Spain	January 2018-July 2019	124	38.3 ± 9.3 (cases) 35.8 ± 10.4 (control)	Case-control
Li et al. 2020 [44]	Hangzhou, China	January 2015–September 2018	345	34.48 ± 6.35 (cases) 33.58 ± 5.72 (control)	Case-control
Radwan et al., 2020 [45]	Warsaw, Poland	2017–2019	450	31.28 ± 3.52	Cohort
Wang et al., 2021 [46]	NHANES study	2013–2014 and 2015–2016	655	13	Cross-sectional
Czubacka et al., 2021 [47]	Poland	N/A	511	33.30 ± 3.69	Cross-sectional
Jurewicz et al., 2021 [48]	Gdańsk, Poland	January 2017–December 2017	357	26.6 ± 5.5 (cases) 31.2 ± 6.9 (control)	Case-control
Lin et al., 2021 [49]	Guangzhou, China	September 2015–June 2016	106	30.8 ± 4.6 (control) 31.8 ± 4.1 (cases)	Case-control
Aftabsavad et al., 2021 [50]	Tehran, Iran	N/A	80	29.60 ± 3.77 (cases) 29.66 ± 4.26 (control)	Prospective case-control
Nazlı Yenigül et al., 2021 [51]	Ankara, Turkey	April 2019–September 2019	82	29.6 ± 3.2	Prospective cohort
Lazúrová et al., 2021 [52]	Košice, Slovakia	N/A	86	28.5 ± 5.1	Case-control

**Table 2:** Bisphenol-A and general fertility.

Author	Exposure Assessment	Outcome Measures	Confounders	Statistical Analysis	BPA (Mean ± SD) (ng/mL)	Main Findings	Limitations	Strength
Caserta et al., 2013 [18]	Serum BPA concentration	Levels of gene expression of nuclear receptors: Estrogen receptors (ERα, Erβ), androgen receptor (AR), pregnane X receptor (PXR), aryl hydrocarbon receptor (AhR).	Age, BMI	Pearson’s test	N/A	Higher BPA levels in infertile women than controls (*p* < 0.01). Infertile patients showed gene expression levels of ERα, ERβ, AR and PXR significantly higher than controls (*p* < 0.05). In infertile women, a positive association was found between BPA levels and ERα, ERβ, AR, AhR and PXR expression (*p* < 0.0005)	Small sample size	Ν/A
Souter et al., 2013 [19]	Urinary BPA concentration	Ovarian reserve: Antral follicle count (AFC), day-3 FSH, and ovarian volume (OV)	Age	Multivariable linear regression	Antral follicle count: 1.6 ± 2.0 Day 3 FSH: 1.7± 2.1 Ovarian volume: 1.5 ± 1.8	Decrease in AFC of 12%, 22%, and 17%, in the 2nd, 3rd and 4th SG–BPA quartile compared to the 1st quartile (*p* < 0.001) No significant associations between quartiles of BPA urinary concentrations and either day-3 FSH serum levels (*p* = 0.64) or OV (*p* = 0.8)	The method of quantification was of low sensitivity, lack of data from the prenatal period, the sites targeted by BPA and critical time periods can be multiple (not only oocytes), AMH was not measured, co-exposures to other select chemicals were not accounted for and exposure to BPA may be reflective of other unknown lifestyle factors that might affectovarian reserves.	Diverse patient population, all urine samples were collected and processed under one protocol, BPA and FSH measured by the same laboratory using the same assay. AFC was determined by infertility specialists only, all working at the same center and following the same guidelines to minimize between-operator variability.
Mínguez-Alarcón et al. 2015 [23]	Urinary BPA concentration	Endometrial wall thickness, peak E2 levels, proportion of high-quality embryos, fertilization rates, implantation rates, clinical pregnancy rates, live birth rates	Age, BMI, race, smoking status, initial infertility diagnosis, number of embryos transferred	Multivariable generalized linear mixed models with random intercepts	1.87 ± 1.57	Urinary BPA concentrations were not associated with endometrial wall thickness (*p* = 0.63), peak E2 levels (*p* = 0.31), proportion of high-quality embryos (*p* = 0.61) or fertilization rates (*p* = 0.51) No associations between urinary BPA concentrations and number of embryos transfer (*p* = 0.09), implantation rate, clinical pregnancy or live birth rates per initiated cycle or per embryo transfer	Single-spot urine sample, difficulties in extrapolating the findings to the general population	Prospective study design, comprehensive adjustment of possible confounding variables and the adequate power (80%) of the study
Miao et al., 2015 [24]	Creatinine adjusted urinary BPA concentration	Concentrations of serum reproductive hormones: FSH, E2, PRL, and PROG	Occupational exposure, age, passive smoking, menstrual regularity (yes/no) and study site, menstrual phases	Multiple linear regression	Cases: 2.22 Control: 0.09	Increased urinary BPA significantly associated with higher PRL (*p* = 0.02) and PROG levels (*p* = 0.01). Positive association between urine BPA and E2 among exposed workers (*p* = 0.05) and a statistically significant inverse association between urine BPA and FSH among the unexposed group (*p* = 0.006).	No restriction of menstrual phases, single-spot urine sample in the un-exposed and twice in the exposed group, BMI not collected/included.	N/A
Zhou et al., 2016 [27]	Creatinine adjusted urinary BPA concentration	AFC, anti-Müllerian hormone (AMH), day-3 FSH and inhibin B (INHB)	Age, BMI, and household income	Multivariable linear regression models	N/A	An inverse association between urinary BPA concentration and AFC was reported. A unit increase in BPA was associated with a significant decrease, of 0.32, in AFC (*p* = 0.01). BPA was also negatively associated with AMH and day-3 FSH levels but neither of them were statistically significant. BPA was not associated with INHB.	Single-spot urine samples, cannot interpret results for the general population	Largest human study to evaluate the associations between environmental exposure to BPA and ovarian reserves.
Pednekar et al., 2019 [32]	Serum BPA concentration	BPA levels in fertile and infertile women	Age, height, weight, BMI and age at menarche	Two-sided independent samples *t*-test	Cases: 4.66 + 3.52 Control: 2.64 + 3.99	The plasma BPA levels were significantly (*p* < 0.05) higher in infertile women compared to fertile women.	Small sample size	N/A
Özel et al., 2019 [33]	Serum BPA concentration	Premature Ovarian Insufficiency (POI) diagnosis	Age, BMI, and tobacco smoking	Student’s t-test and by Mann–Whitney U-test	Cases: 2.85 ± 1.21 Control: 2.46 ± 1.69	No significant difference was observed between the groups regarding serum BPA concentrations (*p* = 0.31)	Small study population, not representative for all women	Carefully recruited study population
Kim et al., 2019 [35]	Urinary, plasma, follicular fluid and semen BPA concentration	Pregnancy rates, presence of good-quality embryos, the proportion of normally fertilized oocytes, number of retrieved oocytes, peak E2 level, sperm concentration and sperm motility	Age, BMI, ethnicity (Asian or Russian) and AMH	Logistic regression models, multiple linear regression models	Urine specimen: 1.16 Plasma specimen: 0.049 Follicular fluid specimen: 0.040	Female urine SG-adjusted BPA, female plasma BPA, follicular BPA concentrations did not significantly affect the parameters, such as the number of retrieved oocytes and peak E2 level, as well as outcomes, such as pregnancy, presence of good quality embryos, or the proportion of normally fertilized oocytes	Single-sport urine measurement, substantial measurement error and attenuation of associations, many urine samples were below LOD, difference between assumption for the a priori sample size estimate and actual data analysis, possible residual confounding factors, no data collection of ongoing pregnancy or live birth	Quite large sample size, data provided meaningful information, examination of various body fluids
Arya et al., 2020 [37]	Urinary BPA concentration	Self-reported infertility	Age, race, ethnicity, marital status, education, annual family income, BMI, waist circumference (WC), tobacco use, alcohol use and physical activity, age at first menstrual period, age at first and last birth, gravidity, parity and treatment for a pelvic infection/pelvic inflammatory disease (PID)	Variable cluster analysis, relative risk regression models	Cases: 2.94 Control: 2.84	No association between infertility and individual BPA concentrations. A positive association between infertility and combined exposure of BPA, BP-3 and TCS was reported.	Single-spot urine measurement, self-report measure of infertility	First study reporting the association between non-persistent EDCs and self-reported infertility in women representative of U.S. population, variable cluster analysis to assess the effect of exposure to mixture of the environmental toxins affecting female fertility, comprehensive adjustment for other reproductive and lifestyle confounding factors
Pollock et al., 2020 [38]	Creatinine-adjusted urinary BPA concentration	Serum-sex-steroid hormone concentration E2, progesterone (P4) and testosterone)	Age, BMI, household income, population group, level of education, tobacco smoke exposure and time of day at sample collection (only level of education was considered as a covariate between household income and level of education)	Multivariable regression models	N/A	BPA was associated with lower levels of E2 in adolescents aged 12 to 19 of either sex. No associations between BPA and hormones in adults was observed.	Single-spot urine samples, not measured emerging chemicals, such as bisphenol analogues	Data representative of the general population
Park et al., 2020 [39]	Urinary BPA concentration	AMH, AFC and OV	Socioeconomic status, alcohol intake, smoking, exercise, medical history, gynecologic history, diet, environmental factors, height, weight, BMI, birth-control-pill usage.	Linear regression, logistic regression analysis	Cases: 0.189 ± 0.217 Control: 0.158 ± 0.108	Urinary bisphenol A (BLA) level significantly higher in the DOR group with anti-Müllerian hormone lower than 25th percentile, no significant correlation between EDCs and AFCs or OV, increase in infertility in BPA level ≥ 90th-percentile group (*p* < 0.005).	Relatively small sample size, short half-life of BPA, exposure to a mixture of other different chemicals simultaneously, lack of stringent confirmation of medical history of volunteers	Representative of general population (volunteers, not patients)
Milczarek-Banach et al., 2020 [40]	Serum and urine concentration of BPA and its 10 analogs	TSH, free thyroxine (fT4), thyroid-peroxidase antibody and thyroglobulin antibody concentration in serum samples, thyroid volume	Relatively high LOD, variations in the stability of phenolic compounds (BPs) in fluids	Spearman correlation test	Serum specimen: 0.133 ± 0.280 Urine specimen: 0.330 ± 0.405	Negative correlation between thyroid volume and urine concentration of BPC (*p* = 0.0005), positive correlation between TSH and urine BPC concentrations (*p* = 0.002). Patients with detected urine BPC presented smaller thyroid glands than those with non-detected urine BPC (*p* = 0.0008).	Small sample size, did not consider mixtures of thyroid EDCs and their synergies	Homogenous group of female participants (age, habitat)
Shen et al., 2020 [41]	Urinary BPA concentration	Dose of gonadotropin, number of dominant follicles, oocytes retrieved, E2 levels, endometrial thickness, implantation rates, clinical pregnancy rates, live birth rates	Age, BMI, basic FSH level, basic E2 level, and AFC	Multivariable generalized linear mixed models	N/A	No association of the urinary BPA concentration with the E2 peak level, endometrial wall thickness, number of dominant follicles, or total dose of gonadotropins, fertilization rate, cleavage rate, and quality of the embryos. Association of high urinary BPA levels and a decrease in the number of retrieved oocytes and in the rates of clinical pregnancy and implantation.	Male partners’ exposure was not considered, no measured urinary BPA concentration on the day of the first frozen ET, non-differential misclassification of exposure	Focus on women with tubal-factor infertility, prospective design
Li et al., 2020 [44]	Urinary BPA levels	LH, FSH, AMH, POI diagnosis	Age at enrollment, age of menarche, higher education, and annual household income	Multinomial logistic regression models	Cases: 0.355 Control: 0.322	Among BPA quartiles, no obvious association was found between BPA levels and the risk of POI (*p* = 0.603). LH was significantly positively associated with BPA levels (*p* = 0.042). FSH and AMH levels showed no tendency toward association with BPA (*p* = 0.941 and *p* = 0.876 for FSH and AMH).	Post-diagnostic assessment of exposure levels, single-spot measurement	Large sample size considering that the morbidity of POI was approximately 1%, adjustment for potential confounding factors
Radwan et al., 2020 [45]	Urinary BPA concentration	Metaphase II (MII) oocyte yield, high-quality embryo, fertilization rate, implantation rate and clinical pregnancy	Age, BMI, smoking status, and infertility diagnosis	Multivariable generalized linear mixed analyses with random intercepts	1.70 ± 2.28	A significant decrease was observed between the urinary concentration of BPA and implantation (*p* = 0.04) and decreased MII oocyte count (*p* = 0.03). There was no association between other examined IVF outcomes: embryo quality, fertilization rate, and clinical pregnancy, and BPA exposure	Cannot be generalized to the general population	Same center, using the same standardized protocol. Detailed questionnaire data on demographics, medical and lifestyle risk factors allowed for control of confounding in the statistical analysis. In addition, all study participants provided at least 1 urine sample per cycle
Wang et al., 2021 [46]	Urinary levels of BPA, BPS, BPF	E2, SHBG, TT/E2	N/A	Multiple linear regression models	Ν/A	BPA was negatively associated with FAI and E2 while positively associated with SHBG and TT/E2 in female adolescents	Single-spot measurement, associations identified in the current study might be twisted as variations in urinary chemicals and serum sex hormones may exist in predefined sex/age groups. Furthermore, as only gonadal hormones (E2, TT) and SHBG were measured in NHANES 2013–2016, the observed association between measured sex-hormone indicators and bisphenols cannot completely depict the effects of BPA, BPF and BPS exposure on the HPG axis. No simultaneous EDC exposure considered	Nationally representative population
Czubacka et al., 2021 [47]	Urinary BPA levels	E2, FSH, AFC, AMH	Infertility diagnosis, age, BMI, smoking	Multivariable linear regression	1.60 ± 2.15	A negative association between BPA urinary concentrations and AMH (*p* = 0.02) and AFC (*p* = 0.03) levels was found. Exposure to BPA was not related to other examined parameters of ovarian reserves (FSH, E2).	The study was conducted on women from an infertility clinic, which may limit the ability to generalize the results to the general population, single-sport urine measurement	One center, same standardized protocol, confounding control. BPA, FSH, AMH, E2 concentrations were each determined by the same laboratory. AFC assessed by trained gynecologists. All the studies were performed at the beginning of follicular phase, most often between 2 and 4 days of the cycle, while menstrual period occurred spontaneously.
Lin et al., 2021 [49]	Urinary BPA levels	Oocyte retrieval rate, maturation rate and embryo implantation rate, peak E2 level	N/A	One-way ANOVA	1.372 ± 1.869	The oocyte retrieval rate, maturation rate and embryo implantation rate significantly decreased with the higher level of urinary BPA concentration. Peak E2 level was lower in high-BPA group, but no statistical significance could be observed. The total antral follicle count was slightly lower in high-BPA-exposure group (16.8 ± 9.0 vs. 13.7 ± 6.7) (*p* = 0.076)	N/A	N/A
Aftabsavad et al., 2021 [50]	follicular fluid BPA levels	AFC, AMH, No. oocyte, No. MII, oocyte quality, total embryos, clinical pregnancy ratio, ICAM-1- and HLA-G-gene expressions	N/A	Univariate and backward multiple linear regression	Cases: 4.73 ± 2.23 Control: 1.56 ± 1.33	CAM-1 and HLA-G genes as well as protein expressions in group 1 (POR—without use of plastic containers) were up-regulated compared to the second group (*p*< 0.05). DNA-methylation status in group 1 was decreased compared to the other group (*p* < 0.05). The concentration of BPA in the follicular fluid of group 1 was lower compared to the second group (*p* < 0.05). The oocyte quality and clinical pregnancy ratio were significantly higher in group 1 than in the other groups (*p* < 0.05).	Sample collection and limitated use of laboratory facilities due to COVID-19	N/A
Nazli Yenigul et al., 2021 [51]	Urinary and serum maternal fluid and follicular fluid	Embryo grade, implantation rate, clinical pregnancy, miscarriage rate and live birth rate	Diet, stress, other supplements	Spearman’s correlation analysis.	Blood specimen Cases: 22.6 ± 17.1 Control: 10.2 ± 7.7 Follicular Fluid specimen Cases: 14.4 ± 10.0 Control: 7.4 ± 6.9 Urine specimen Cases: 8.1 ± 3.9 Control: 7.0 ± 3.0	Women who had grade-1 embryos transferred had lower BPA levels (*p* = 0.003). Serum and follicular-fluid BPA levels were significantly higher in women who failed to achieve clinical pregnancy. A negative correlation was found between follicular fluid and serum BPA values and E2 values on HCG day in the group who failed to achieve clinical pregnancy (*p* = 0.002 and *p* = 0.048)	Small number of study participants, single-point urine and blood BPA sample	Clearly identifiable source of BPA levels. The IVF outcomes, BPA levels in body fluids and the information on the primary source of chronic BPA exposure as plastic bottled water were analyzed together

**Table 3:** Bisphenol-A (BPA) and polycystic ovary syndrome (PCOS).

Author, Year	BPA Sample Assessment	Outcome Measures	Confounders	Statistical Analysis	BPA (Mean) (ng/mL)	Main Findings	Limitations	Strengths
Tarantino et al., 2013 [16]	Serum BPA concentration	Homeostasis-model assessment of insulin resistance (HoMA-IR), testosterone, sex hormone-binding globulin (SHBG), free androgen index (FAI)	Age, BMI, waist circumference	Multiple linear regression analysis	N/A	BPA higher in PCOS women than in controls (*p* < 0.0001). Higher BPA in PCOS women were associated with higher grades of insulin resistance (*p* = 0.0003), HS (*p* = 0.027), FAI (*p* = 0.025) and larger spleen size (*p* < 0.0001)	Ν/A	Ν/A
Vagi et al., 2014 [21]	Creatinine-adjusted urine BPA concentration	Diagnosis of PCOS	Age, BMI, race	Multivariate logistic regression analysis	Cases: 1.6 Control: 1.9	No association between PCOS and BPA (*p* > 0.05).	Small sample size, single-spot urine specimen, larger proportion of case-patients were of white race compared to controls, PCOS patients were younger and had significantly higher BMI than controls	N/A
Akin et al., 2015 [22]	Serum BPA concentration	Diagnosis of PCOS (modified Rotterdam criteria), insulin resistance	Age, BMI	Multivariate linear regression analysis with backward elimination	Cases: 1.1 Control: 0.8	Higher BPA in adolescents with PCOS than controls, independent of obesity (*p* = 0.001). BPA concentrations were also significantly correlated with androgen levels (*p* < 0.05)	No simultaneous EDC exposure considered	Large series of adolescent population sample with PCOS.
Vahedi et al., 2016 [25]	Serum BPA concentration	Fasting blood sugar (FBS), triglyceride, cholesterol, HDL and LDL levels, thyroid-stimulating hormone (TSH) concentration and LH:FSH ratio (serum levels)	Age, BMI	Two independent sample *t*-tests	Cases: 0.48 ± 0.08 Control: 0.16 ± 0.04	In PCOS women, BPA level was higher than in healthy women (*p* < 0.001). Significant differences in levels of triglycerides (*p* < 0.001), cholesterol (*p* = 0.045), TSH (*p* < 0.05) and LH: FSH ratio (*p* < 0.001)	N/A	N/A
Rashidi et al., 2017 [29]	Urinary BPA concentration	Diagnosis of PCOS	Age, BMI, parity, menstrual irregularity, history of abortion, education	Logistic regression analysis	Cases: 1.79 ± 8 Control:0.81 ±2.92	BPA was significantly associated with PCOS (*p* < 0.001)	No creatinine measurement/adjustment, single-spot BPA measurement, no sufficient data about the hormonal profile of patients	Measurement of BPA in urine specimens
Gu et al., 2018 [30]	Urinary BPA concentration	Risk of diagnosis of PCOS	BMI	Binary logistic regression model	N/A	No significant relationships between PCOS and urinary BPA levels (*p* > 0.05).	Small study population	N/A
Konieczna et al., 2018 [31]	Serum BPA concentration	Diagnosis of PCOS, serum total testosterone (TST), FAI	Age, BMI, waist circumference, serum glucose, insulin concentration and lipids	One-way analysis of variance (ANOVA)	Cases: 0.202 Control: 0.154	Women with PCOS had significantly higher serum BPA concentrations than healthy controls (*p* = 0.035). In women with PCOS, serum BPA concentrations correlated positively with FAI and TST concentration (*p* = 0.049, *p* = 0.004)	Serum BPA concentrations, TST concentrations were measured using an automated electrochemiluminescence immunoassay	Both clinical and laboratory diagnosis of PCOS
Akgul et al., 2019 [36]	Urinary BPA concentration	Ultrasonographic findings of PCOS, serum follicle-stimulating hormone (FSH), luteinizing hormone (LH), estradiol (E2), prolactin (PRL), thyrotropin, fT3 and fT4, serum dehydroxyepiandrosterone sulfate, 17-hydroxyprogesterone (17OH-P), TST, SHBG, fasting insulin, insulin resistance	Obesity	Spearman’s correlation test	Cases: 15.89 ± 1.16 Control: 7.3 ± 1.38	BPA significantly associated with PCOS (*p* = 0.016). In adolescents with PCOS, BPA was significantly correlated with polycystic morphology on ultrasound but not with obesity, androgen levels or other metabolic parameters	Small number of patients, single-spot urine specimen for BPA, no simultaneous EDCs exposure considered	N/A
Lazúrová et al., 2021 [52]	Creatinine-adjusted urinary BPA concentration	Diagnosis of PCOS	Age, BMI	Linear regression analysis	Cases: 6.1 ± 0.9 Control: 1.6 ± 0.6	PCOS women had significantly higher U-BPA as compared with the control group (*p* = 0.0001), high levels of U-BPA are associated with higher TST (*p* = 0.029) and HOMA IR (*p* = 0.037), lower serum estrone (*p* = 0.05),E2 (*p* = 0.0126), FSH (*p* = 0.0056) and FAI (*p* = 0.0088)	Smaller group of control subjects than PCOS women, measurement of steroid hormones by immunoassay, non-use of HPLC-MS	Large group of PCOS women, measurement of urinary concentration of BPA
Jurewicz et al., 2021 [48]	Serum BPA, BPS, BPF levels	Serum total cholesterol (TCh), HDL-cholesterol (HDL-C), triglycerides (TG), fasting glucose, FSH, LH, TSH, PRL, E2, TST, dehydroepiandrosterone sulphate (DHEA-S), progesterone (PRG), 17OH-P, and insulin concentrations	Age, education, BMI, income, smoking, alcohol consumption	Logistic regression models	Cases: 0.46 Control: 0.33	In women with PCOS, BPS concentrations were significantly higher compared to the control subjects (*p* = 0.023). Serum BPA and BPF concentrations did not differ between the studied groups. Negative correlation between serum BPA and HOMA IR (*p* = 0.001) and TST (*p* = 0.006) in women with PCOS	Single-spot serum sample, women with PCOS were significantly younger compared to the controls subjects and, therefore, may have had a lower life-time exposure to the studied EDC	N/A

**Table 4:** Bisphenol-A (BPA) and endometriosis.

Author	Exposure Assessment	Outcome Measures	Confounders	Statistical Analysis	BPA (Mean) (ng/mL)	Main Findings	Limitations	Strength
Buck Louis et al., 2013 [17]	Creatine-adjusted urinary BPA concentration	Diagnosis of endometriosis	Age, BMI, parity conditional on gravidity (never pregnant, pregnant without births, pregnant with births)	Logistic regression analysis	Operative cohort: Cases: 1.45 Control: 1.66 Population cohort: Cases: 4.19 Control: 1.65	No relationship between BPA and endometriosis was reported (*p* > 0.05). A statistically significant association emerged when adjusting for parity along with other relevant covariates in the population cohort	Collection of urine samples across women’s menstrual cycles, relatively short interval between quantification of urinary chemicals and diagnosis, inability to detect endometriosis stages 1–2 in the population cohort, exploratory nature of the analysis	Novel study design (both an operative and population cohort), measurement of BPA in urine
Upson et al., 2014 [20]	Creatinine-adjusted urinary BPA concentration	Diagnosis of endometriosis	Age, education, alcohol consumption, smoking status and race	Unconditional logistic regression analysis	N/A	No statistically significant association between total urinary BPA concentrations and endometriosis was found overall (*p* > 0.05). Increased urinary BPA seems to be associated with an increased risk of non-ovarian pelvic endometriosis, but not ovarian endometriosis	Single-spot urine sample, no surgical confirmation of the absence of disease in controls	WREN study size and extensive information collected in the study, population-based sampling framework, minimal external contamination of samples and degradation of conjugated BPA during storage
Simonelli et al., 2016 [26]	Urinary and peritoneal fluid BPA concentration	Diagnosis of endometriosis	Environmental factors, smoking, use of food plastic boxes, occupational exposure	Use of t-test for independent variables, Mann–Whitney U-test	N/A	A statistically significant association between BPA exposure and endometriosis was reported (*p* = 0.001). No significant correlation was found between BPA U and BPA *p* levels (*p* > 0.05)	The odds ratios relating endometriosis to occupation and environmental/lifestyle factors do not account for potential confounders	N/A
Rashidi et al., 2017 [28]	Urinary BPA concentration	Diagnosis of endometrioma	Age, parity, BMI and educational status	Mann–Whitney U-test, logistic regression analysis	Cases: 5.53 ± 3.46 Control: 1.42 ± 1.56	A positive association between urinary BPA concentrations and endometrioma was reported (*p* < 0.0001)	Non-ovarian endometriosis could not be detected in the control group, no creatinine measurement, one-time urine sample BPA detection, did not evaluate BPA exposure before disease onset compared with levels at the time of diagnosis or surgery	Measurement of BPA in urine specimens
Moreira Fernandez et al., 2019 [34]	Urine BPA concentration	Diagnosis of endometriosis	Food habits, beverages and cigarette consumption, duration of menstrual bleeding, age at menarche, positive history of endometriosis in the family, use of oral contraceptives and hormones, BMI, measurement of creatinine	Chi-square test and Odds Ratio.	N/A	No association was observed at BPA metabolite concentration in the urine and endometriosis samples (*p* = 0.502)	Window between the environmental chemical exposure and disease manifestation	Adequate selection of biological fluid, analysis of the use of metabolites instead of the use of parent compounds, strict inclusion criteria
Wen et al., 2020 [42]	Creatinine-adjusted urinary BPA concentration	MMP2 and MMP9 serum concentration	Co-exposure to other EDCs, smoking status, BMI, alcohol consumption, age, childbearing history	Unconditional logistic regression models	N/A	Creatinine-adjusted urinary BPA concentrations were positively correlated with serum MMP2, MMP9 levels, and the risk of peritoneal EMs (*p* = 0.030).	Single-spot urine sample, urine sample after the onset of Endometriomas (Ems), relatively small sample size for EMs, especially for peritoneal Ems.	Exclusion of Ems cases (undiagnosed Ems in the unscreened general population) in controls based on B-ultrasound
Peinado et al., 2020 [43]	Creatinine-adjusted urinary BPA, BPS and BPF concentration	Risk for endometriosis, quantification of TBARS in urine	Age, BMI, parity, residence, educational level, occupational status, smoking habits	Multivariate logistic and linear regression analyses	Cases: 5.5 ± 1.1 Control: 3.0 ± 1.2	BPA concentrations associated with an increased risk of endometriosis (*p* = 0.089). TBARS concentrations showed a close-to-significant relationship with increased endometriosis risk. The association between endometriosis risk and concentrations of BPA and Σbisphenols was only statistically significant for women in the highest TBARS tertile	Limited sample size, no simultaneous EDCs exposure considered, collection of urine samples across women’s menstrual cycles.	Visual and histologic confirmation of the presence of endometriosis in cases and its absence in controls, first evaluation of exposure to BPA analogues BPS and BPF, novel evidence of a mediating role for lipid peroxidation

## Data Availability

Data is contained within the article.

## References

[1] Conforti A., Mascia M., Cioffi G., De Angelis C., Coppola G., De Rosa P., Pivonello R., Alviggi C., De Placido G. (2018). Air pollution and female fertility: A systematic review of literature. Reprod. Biol. Endocrinol..

[2] Kassotis C.D., Vandenberg L.N., Demeneix B.A., Porta M., Slama R., Trasande L. (2020). Endocrine-disrupting chemicals: Economic, regulatory, and policy implications. Lancet Diabetes Endocrinol..

[3] Alkema L., Chou D., Hogan D., Zhang S., Moller A.B., Gemmill A., Fat D.M., Boerma T., Temmerman M., Mathers C. (2016). Global, regional, and national levels and trends in maternal mortality between 1990 and 2015, with scenario-based projections to 2030: A systematic analysis by the UN Maternal Mortality Estimation Inter-Agency Group. Lancet.

[4] Sharrow D., Hug L., You D., Alkema L., Black R., Cousens S., Croft T., Gaigbe-Togbe V., Gerland P., Guillot M. (2022). Global, regional, and national trends in under-5 mortality between 1990 and 2019 with scenario-based projections until 2030: A systematic analysis by the UN Inter-agency Group for Child Mortality Estimation. Lancet Glob. Health.

[5] Prevention C.F.D.C.A. Key Statistics from the National Survey of Family Growth—I Listing. https://www.cdc.gov/nchs/nsfg/key_statistics/i.htm.

[6] World Health Organization Infertility. https://www.who.int/news-room/fact-sheets/detail/infertility.

[7] Vandenberg L.N., Hauser R., Marcus M., Olea N., Welshons W.V. (2007). Human exposure to bisphenol A (BPA). Reprod. Toxicol..

[8] Healy B.F., English K.R., Jagals P., Sly P. (2015). Bisphenol A exposure pathways in early childhood: Reviewing the need for improved risk assessment models. J. Expo. Sci. Environ. Epidemiol..

[9] Ma Y., Liu H., Wu J., Yuan L., Wang Y., Du X., Wang R., Marwa P.W., Petlulu P., Chen X. (2019). The adverse health effects of bisphenol A and related toxicity mechanisms. Environ. Res..

[10] Tarafdar A., Sirohi R., Balakumaran P.A., Reshmy R., Madhavan A., Sindhu R., Binod P., Kumar Y., Kumar D., Sim S.J. (2022). The hazardous threat of Bisphenol A: Toxicity, detection and remediation. J. Hazard. Mater..

[11] Chevalier N., Fénichel P. (2015). Bisphenol A: Targeting metabolic tissues. Rev. Endocr. Metab. Disord..

[12] Lee H.J., Chattopadhyay S., Gong E.-Y., Ahn R.S., Lee K. (2003). Antiandrogenic effects of bisphenol A and nonylphenol on the function of androgen receptor. Toxicol. Sci..

[13] Nadal A., Fuentes E., Ripoll C., Villar-Pazos S., Castellano-Muñoz M., Soriano S., Martinez-Pinna J., Quesada I., Alonso-Magdalena P. (2018). Extranuclear-initiated estrogenic actions of endocrine disrupting chemicals: Is there toxicology beyond paracelsus?. J. Steroid. Biochem. Mol. Biol..

[14] Ziv-Gal A., Flaws J.A. (2016). Evidence for bisphenol A-induced female infertility: A review (2007–2016). Fertil. Steril..

[15] Vandenberg L.N., Ehrlich S., Belcher S.M., Ben-Jonathan N., Dolinoy D.C., Hugo E.R., Hunt A.P., Newbold R.R., Rubin B.S., Saili K.S. (2013). Low dose effects of bisphenol A. Endocr. Disruptors.

[16] Tarantino G., Valentino R., Di Somma C., D’Esposito V., Passaretti F., Pizza G., Brancato V., Orio F., Formisano P., Colao A. (2013). Bisphenol A in polycystic ovary syndrome and its association with liver-spleen axis. Clin. Endocrinol..

[17] Louis G.M.B., Peterson C.M., Chen Z., Croughan M., Sundaram R., Stanford J., Varner M.W., Kennedy A., Giudice L., Fujimoto V.Y. (2013). Bisphenol A and phthalates and endometriosis: The Endometriosis: Natural History, Diagnosis and Outcomes Study. Fertil Steril.

[18] Caserta D., Ciardo F., Bordi G., Guerranti C., Fanello E., Perra G., Borghini F., La Rocca C., Tait S., Bergamasco B. (2013). Correlation of Endocrine Disrupting Chemicals Serum Levels and White Blood Cells Gene Expression of Nuclear Receptors in a Population of Infertile Women. Int. J. Endocrinol..

[19] Souter I., Smith K.W., Dimitriadis I., Ehrlich S., Williams P.L., Calafat A.M., Hauser R. (2013). The association of bisphenol-A urinary concentrations with antral follicle counts and other measures of ovarian reserve in women undergoing infertility treatments. Reprod. Toxicol..

[20] Upson K., Sathyanarayana S., De Roos A.J., Koch H.M., Scholes D., Holt V.L. (2014). A population-based case–control study of urinary bisphenol A concentrations and risk of endometriosis. Hum. Reprod..

[21] Vagi S.J., Azziz-Baumgartner E., Sjödin A., Calafat A.M., Dumesic D., Gonzalez L., Kato K., Silva M.J., Ye X., Azziz R. (2014). Exploring the potential association between brominated diphenyl ethers, polychlorinated biphenyls, organochlorine pesticides, perfluorinated compounds, phthalates, and bisphenol a in polycystic ovary syndrome: A case–control study. BMC Endocr. Disord..

[22] Akın L., Kendirci M., Narin F., Kurtoglu S., Saraymen R., Kondolot M., Koçak S., Elmalı F., Elmali F. (2015). The endocrine disruptor bisphenol A may play a role in the aetiopathogenesis of polycystic ovary syndrome in adolescent girls. Acta Paediatr..

[23] Mínguez-Alarcón L., Gaskins A.J., Chiu Y.H., Williams P.L., Ehrlich S., Chavarro J.E., Petrozza J.C., Ford J.B., Calafat A.M., Hauser R. (2015). Urinary bisphenol A concentrations and association with in vitro fertilization outcomes among women from a fertility clinic. Hum. Reprod..

[24] Miao M., Yuan W., Yang F., Liang H., Zhou Z., Li R., Gao E., Li D.-K. (2015). Associations between Bisphenol A Exposure and Reproductive Hormones among Female Workers. Int. J. Environ. Res. Public Health.

[25] Vahedi M., Saeedi A., Poorbaghi S.L., Sepehrimanesh M., Fattahi M.R. (2016). Metabolic and endocrine effects of bisphenol A exposure in market seller women with polycystic ovary syndrome. Environ. Sci. Pollut. Res..

[26] Simonelli A., Guadagni R., De Franciscis P., Colacurci N., Pieri M., Basilicata P., Pedata P., Lamberti M., Sannolo N., Miraglia N. (2017). Environmental and occupational exposure to bisphenol A and endometriosis: Urinary and peritoneal fluid concentration levels. Int. Arch. Occup. Environ. Health.

[27] Zhou W., Fang F., Zhu W., Chen Z.-J., Du Y., Zhang J. (2017). Bisphenol A and Ovarian Reserve among Infertile Women with Polycystic Ovarian Syndrome. Int. J. Environ. Res. Public Health.

[28] Eslami B., Rashidi B.H., Amanlou M., Lak T.B., Ghazizadeh M. (2017). A case-control study of bisphenol A and endometrioma among subgroup of Iranian women. J. Res. Med. Sci..

[29] Rashidi B.H., Amanlou M., Lak T.B., Ghazizadeh M., Haghollahi F., Bagheri M., Eslami B. (2017). The Association Between Bisphenol A and Polycystic Ovarian Syndrome: A Case-Control Study. Acta Med. Iran..

[30] Gu J., Yuan T., Ni N., Ma Y., Shen Z., Yu X., Shi R., Tian Y., Zhou W., Zhang J. (2019). Urinary concentration of personal care products and polycystic ovary syndrome: A case-control study. Environ. Res..

[31] Konieczna A., Rachoń D., Owczarek K., Kubica P., Kowalewska A., Kudłak B., Wasik A., Namieśnik J. (2018). Serum bisphenol A concentrations correlate with serum testosterone levels in women with polycystic ovary syndrome. Reprod. Toxicol..

[32] Pednekar P.P., Gajbhiye R.K., Patil A.D., Surve S.V., Datar A.G., Balsarkar G.D., Chuahan A.R., Vanage G.R. (2018). Estimation of plasma levels of bisphenol-A & phthalates in fertile & infertile women by gas chromatography-mass spectrometry. Indian J. Med. Res..

[33] Özel Ş., Tokmak A., Aykut O., Aktulay A., Hançerlioğulları N., Engin Ustun Y. (2019). Serum levels of phthalates and bisphenol-A in patients with primary ovarian insufficiency. Gynecol. Endocrinol..

[34] Fernandez M.A.M., Cardeal Z.L., Carneiro M.M., André L.C. (2019). Study of possible association between endometriosis and phthalate and bisphenol A by biomarkers analysis. J. Pharm. Biomed. Anal..

[35] Kim H.-K., Ko D.-H., Lee W., Kim K.-R., Chun S., Song J., Min W.-K. (2021). Body fluid concentrations of bisphenol A and their association with in vitro fertilization outcomes. Hum. Fertil..

[36] Akgül S., Sur U., Düzçeker Y., Balcı A., Kızılkan M.P., Kanbur N., Bozdağ G., Erkekoğlu P., Gumus E., Kocer-Gumusel B. (2019). Bisphenol A and phthalate levels in adolescents with polycystic ovary syndrome. Gynecol. Endocrinol..

[37] Arya S., Dwivedi A.K., Alvarado L., Kupesic-Plavsic S. (2020). Exposure of U.S. population to endocrine disruptive chemicals (Parabens, Benzophenone-3, Bisphenol-A and Triclosan) and their associations with female infertility. Environ. Pollut..

[38] Pollock T., Arbuckle T.E., Guth M., Bouchard M.F., St-Amand A. (2021). Associations among urinary triclosan and bisphenol A concentrations and serum sex steroid hormone measures in the Canadian and U.S. Populations. Environ. Int..

[39] Park S.Y., Jeon J.H., Jeong K., Chung H.W., Lee H., Sung Y.-A., Ye S., Ha E.-H. (2021). The Association of Ovarian Reserve with Exposure to Bisphenol A and Phthalate in Reproductive-aged Women. J. Korean Med. Sci..

[40] Milczarek-Banach J., Rachoń D., Bednarczuk T., Myśliwiec-Czajka K., Wasik A., Miśkiewicz P. (2021). Exposure to Bisphenol A Analogs and the Thyroid Function and Volume in Women of Reproductive Age—Cross-Sectional Study. Front. Endocrinol..

[41] Shen J., Kang Q., Mao Y., Yuan M., Le F., Yang X., Xu X., Jin F. (2020). Urinary bisphenol A concentration is correlated with poorer oocyte retrieval and embryo implantation outcomes in patients with tubal factor infertility undergoing in vitro fertilisation. Ecotoxicol. Environ. Saf..

[42] Wen X., Xiong Y., Jin L., Zhang M., Huang L., Mao Y., Zhou C., Qiao Y., Zhang Y. (2020). Bisphenol A Exposure Enhances Endometrial Stromal Cell Invasion and Has a Positive Association with Peritoneal Endometriosis. Reprod. Sci..

[43] Peinado F.M., Lendínez I., Sotelo R., Iribarne-Durán L.M., Fernández-Parra J., Vela-Soria F., Olea N., Fernández M.F., Freire C., León J. (2020). Association of Urinary Levels of Bisphenols A, F, and S with Endometriosis Risk: Preliminary Results of the EndEA Study. Int. J. Environ. Res. Public Health.

[44] Li C., Cao M., Qi T., Ye X., Ma L., Pan W., Luo J., Chen P., Liu J., Zhou J. (2021). The association of bisphenol A exposure with premature ovarian insufficiency: A case–control study. Climacteric.

[45] Radwan P., Wielgomas B., Radwan M., Krasiński R., Klimowska A., Kaleta D., Jurewicz J. (2020). Urinary bisphenol A concentrations and in vitro fertilization outcomes among women from a fertility clinic. Reprod. Toxicol..

[46] Wang Y., Aimuzi R., Nian M., Zhang Y., Luo K., Zhang J. (2021). Bisphenol A substitutes and sex hormones in children and adolescents. Chemosphere.

[47] Czubacka E., Wielgomas B., Klimowska A., Radwan M., Radwan P., Karwacka A., Kałużny P., Jurewicz J. (2021). Urinary Bisphenol A Concentrations and Parameters of Ovarian Reserve among Women from a Fertility Clinic. Int. J. Environ. Res. Public Health.

[48] Jurewicz J., Majewska J., Berg A., Owczarek K., Zajdel R., Kaleta D., Wasik A., Rachoń D. (2021). Serum bisphenol A analogues in women diagnosed with the polycystic ovary syndrome—Is there an association?. Environ. Pollut..

[49] Lin M., Hua R., Ma J., Zhou Y., Li P., Xu X., Yu Z., Quan S. (2021). Bisphenol A promotes autophagy in ovarian granulosa cells by inducing AMPK/mTOR/ULK1 signalling pathway. Environ. Int..

[50] Aftabsavad S., Noormohammadi Z., Moini A., Karimipoor M. (2021). Effect of bisphenol A on alterations of ICAM-1 and HLA-G genes expression and DNA methylation profiles in cumulus cells of infertile women with poor response to ovarian stimulation. Sci. Rep..

[51] Yenigül N.N., Dilbaz S., Dilbaz B., Kaplanoğlu I., Güçel F., Aldemir O., Baser E., Ozelci R., Tekin O.M. (2021). The effect of plastic bottled water consumption on outcomes of ICSI cycles undertaken for unexplained infertility. Reprod. Biomed. Online.

[52] Lazúrová Z., Figurová J., Hubková B., Mašlanková J., Lazúrová I. (2021). Urinary bisphenol A in women with polycystic ovary syndrome—A possible suppressive effect on steroidogenesis?. Horm. Mol. Biol. Clin. Investig..

[53] March W.A., Moore V.M., Willson K.J., Phillips D.I., Norman R.J., Davies M.J. (2010). The prevalence of polycystic ovary syndrome in a community sample assessed under contrasting diagnostic criteria. Hum. Reprod..

[54] Dumesic D.A., Oberfield S.E., Stener-Victorin E., Marshall J.C., Laven J.S., Legro R.S. (2015). Scientific Statement on the Diagnostic Criteria, Epidemiology, Pathophysiology, and Molecular Genetics of Polycystic Ovary Syndrome. Endocr. Rev..

[55] Moran L.J., Norman R., Teede H.J. (2015). Metabolic risk in PCOS: Phenotype and adiposity impact. Trends Endocrinol. Metab..

[56] Trikudanathan S. (2015). Polycystic ovarian syndrome. Med. Clin. North Am..

[57] Diamanti-Kandarakis E., Christakou C., Marinakis E. (2012). Phenotypes and enviromental factors: Their influence in PCOS. Curr. Pharm. Des..

[58] Wijeyaratne C.N., Seneviratne R.D.A., Dahanayake S., Kumarapeli V., Palipane E., Kuruppu N., Yapa C., Balen A.H. (2011). Phenotype and metabolic profile of South Asian women with polycystic ovary syndrome (PCOS): Results of a large database from a specialist Endocrine Clinic. Hum. Reprod..

[59] Giudice L.C. (2010). Clinical practice. Endometriosis. N. Engl. J. Med..

[60] Shafrir A., Farland L., Shah D., Harris H., Kvaskoff M., Zondervan K., Missmer S. (2018). Risk for and consequences of endometriosis: A critical epidemiologic review. Best Pract. Res. Clin. Obstet. Gynaecol..

[61] Koninckx P.R., Fernandes R., Ussia A., Schindler L., Wattiez A., Al-Suwaidi S., Amro B., Al-Maamari B., Hakim Z., Tahlak M. (2021). Pathogenesis Based Diagnosis and Treatment of Endometriosis. Front. Endocrinol..

[62] Bloom M.S., Kim D., Vom Saal F.S., Taylor J.A., Cheng G., Lamb J.D., Fujimoto V.Y. (2011). Bisphenol A exposure reduces the estradiol response to gonadotropin stimulation during in vitro fertilization. Fertil. Steril..

[63] Ehrlich S., Williams P.L., Missmer S.A., Flaws J.A., Berry K.F., Calafat A.M., Ye X., Petrozza J.C., Wright D., Hauser R. (2012). Urinary bisphenol A concentrations and implantation failure among women undergoing in vitro fertilization. Environ. Health Perspect..

[64] Baker V.L., Rone H.M., Pasta D.J., Nelson H.P., Gvakharia M., Adamson G.D. (2006). Correlation of thyroid stimulating hormone (TSH) level with pregnancy outcome in women undergoing in vitro fertilization. Am. J. Obstet. Gynecol..

[65] Zhou W., Liu J., Liao L., Han S., Liu J. (2008). Effect of bisphenol A on steroid hormone production in rat ovarian theca-interstitial and granulosa cells. Mol. Cell. Endocrinol..

[66] Kechagias K.S., Semertzidou A., Athanasiou A., Paraskevaidi M., Kyrgiou M. (2020). Bisphenol-A and polycystic ovary syndrome: A review of the literature. Rev. Environ. Health.

[67] Mukhopadhyay R., Prabhu N.B., Kabekkodu S.P., Rai P.S. (2022). Review on bisphenol A and the risk of polycystic ovarian syndrome: An insight from endocrine and gene expression. Environ. Sci. Pollut. Res..

[68] Hugo E.R., Brandebourg T.D., Woo J.G., Loftus J., Alexander J.W., Ben-Jonathan N. (2008). Bisphenol A at environmentally relevant doses inhibits adiponectin release from human adipose tissue explants and adipocytes. Environ. Health Perspect..

[69] Song D.K., Hong Y.S., Sung Y.-A., Lee H. (2017). Insulin resistance according to β-cell function in women with polycystic ovary syndrome and normal glucose tolerance. PLoS ONE.

[70] Takeuchi T., Tsutsumi O., Ikezuki Y., Kamei Y., Osuga Y., Fujiwara T., Takai Y., Momoeda M., Yano T., Taketani Y. (2006). Elevated serum bisphenol A levels under hyperandrogenic conditions may be caused by decreased UDP-glucuronosyltransferase activity. Endocr. J..

[71] Yilmaz B.D., Bulun E.S. (2019). Endometriosis and nuclear receptors. Hum. Reprod. Update.

[72] Donnez J., Dolmans M.M. (2021). Endometriosis and Medical Therapy: From Progestogens to Progesterone Resistance to GnRH Antagonists: A Review. J. Clin. Med..

[73] Völkel W., Colnot T., Csanády G.A., Filser J.G., Dekant W. (2002). Metabolism and kinetics of bisphenol a in humans at low doses following oral administration. Chem. Res. Toxicol..

